# The relevance of cerebrospinal fluid α-synuclein levels to sporadic and familial Alzheimer’s disease

**DOI:** 10.1186/s40478-018-0624-z

**Published:** 2018-11-26

**Authors:** Daniel Twohig, Elena Rodriguez-Vieitez, Sigrid B. Sando, Guro Berge, Camilla Lauridsen, Ina Møller, Gøril R. Grøntvedt, Geir Bråthen, Kalicharan Patra, Guojun Bu, Tammie L. S. Benzinger, Celeste M. Karch, Anne Fagan, John C. Morris, Randall J. Bateman, Agneta Nordberg, Linda R. White, Henrietta M. Nielsen, for the Dominantly Inherited Alzheimer Network (DIAN)

**Affiliations:** 1 Department of Biochemistry and Biophysics, Stockholm University, Svante Arrhenius väg 16B, 106 91 Stockholm, Sweden; 2 Department of Neurobiology, Care Sciences and Society, Karolinska Institutet, Stockholm, Sweden; 3 Department of Neurology, University Hospital of Trondheim, Trondheim, Norway; 4 Department of Neuroscience, Norwegian University of Science and Technology, Trondheim, Norway; 5 Department of Neuroscience, Mayo Clinic College of Medicine, Jacksonville, FL USA; 6 Department of Radiology, Washington University School of Medicine, St Louis, MO USA; 7 Department of Psychiatry, Washington University School of Medicine, St Louis, MO USA; 8 Department of Neurology, Washington University School of Medicine, St Louis, MO USA; 9 The Aging Research Center, Karolinska University Hospital, Stockholm, Sweden

**Keywords:** Alzheimer’s disease, Mild cognitive impairment, alpha-synuclein, Biomarkers, APOEε4

## Abstract

Accumulating evidence demonstrating higher cerebrospinal fluid (CSF) α-synuclein (αSyn) levels and αSyn pathology in the brains of Alzheimer’s disease (AD) patients suggests that αSyn is involved in the pathophysiology of AD. To investigate whether αSyn could be related to specific aspects of the pathophysiology present in both sporadic and familial disease, we quantified CSF levels of αSyn and assessed links to various disease parameters in a longitudinally followed cohort (*n* = 136) including patients with sporadic mild cognitive impairment (MCI) and AD, and in a cross-sectional sample from the Dominantly Inherited Alzheimer’s Network (*n* = 142) including participants carrying autosomal dominant AD (ADAD) gene mutations and their non-mutation carrying family members.

Our results show that sporadic MCI patients that developed AD over a period of two years exhibited higher baseline αSyn levels (*p* = 0.03), which inversely correlated to their Mini-Mental State Examination scores, compared to cognitively normal controls (*p* = 0.02). In the same patients, there was a dose-dependent positive association between CSF αSyn and the *APOEε4* allele. Further, CSF αSyn levels were higher in symptomatic ADAD mutation carriers versus non-mutation carriers (*p* = 0.03), and positively correlated to the estimated years from symptom onset (*p* = 0.05) across all mutation carriers. In asymptomatic (Clinical Dementia Rating < 0.5) PET amyloid-positive ADAD mutation carriers CSF αSyn was positively correlated to ^11^C-Pittsburgh Compound-B (PiB) retention in several brain regions including the posterior cingulate, superior temporal and frontal cortical areas. Importantly, *APOEε4*-positive ADAD mutation carriers exhibited an association between CSF αSyn levels and mean cortical PiB retention (*p* = 0.032). In both the sporadic AD and ADAD cohorts we found several associations predominantly between CSF levels of αSyn, tau and amyloid-β_1–40_.

Our results suggest that higher CSF αSyn levels are linked to AD pathophysiology at the early stages of disease development and to the onset of cognitive symptoms in both sporadic and autosomal dominant AD. We conclude that *APOEε4* may promote the processes driven by αSyn, which in turn may reflect on molecular mechanisms linked to the asymptomatic build-up of amyloid plaque burden in brain regions involved in the early stages of AD development.

## Introduction

Intracellular neurofibrillary tau tangles and extracellular amyloid-β (Aβ) plaques are the main neuropathological hallmarks of Alzheimer’s disease (AD), however, at autopsy more than 50% of these patients exhibit concurrent α-synuclein (αSyn) pathology [1, 16, 31]. Conversely, it has been shown that Aβ plaques promote the development of cortical αSyn lesions in individuals with Parkinson’s disease (PD), and up to 50% of PD with dementia patients develop Aβ and tau pathology sufficient for a secondary pathological diagnosis of AD [19, 46]. α-synuclein, a 140 amino-acid protein encoded by the *SNCA* gene, is abundantly expressed neuronal presynaptic terminals [20]. The peptide corresponding to residues 61–95 of αSyn was originally discovered 25 years ago in Aβ plaques and named the non-Aβ component (NAC) [58]. Follow-up studies from the same group further demonstrated NAC immune-reactivity in diffuse Aβ plaques which may result in the formation of mature compact Aβ plaques [33]. Results from these studies suggest that αSyn might be involved in the development of AD from the very early stages of Aβ pathology formation. In support, a recent study demonstrated a positive correlation between cerebrospinal fluid levels of αSyn and Aβ plaque deposition in cognitively normal individuals with subjective memory complaints. The authors speculated that CSF αSyn levels may be related to pathophysiological mechanisms occurring early in the preclinical phase of AD [59].

The relevance of correlating αSyn levels in the CSF to brain parenchymal concentration of αSyn in AD patients is nevertheless controversial due to the lack of autopsy-validated studies assessing αSyn levels in paired CSF and brain tissue samples. Ideally, advancements in radiotracer chemistry could allow for ante-mortem imaging studies in which CSF αSyn concentrations could be correlated to αSyn load in the antemortem brain, similar to PiB-PET correlations between CSF Aβ levels and brain amyloid plaque load [13]. Hence the exact relationship between the low CSF αSyn and brain parenchymal Lewy bodies as occurring in patients with synucleinopathies like PD [68] and dementia with Lewy bodies (DLB) [37] remains unknown. Similarly, the relevance of slightly increased CSF αSyn levels in AD patients [26] to αSyn pathology in the brain is also unclear.

In a study of brains from AD patients devoid of Lewy bodies, intracellular levels of soluble monomeric αSyn were two-fold higher than in controls and significantly correlated to cognitive decline [28]. Potential alterations of CSF αSyn levels in the same subjects were not investigated. Although αSyn can interact with a multitude of cellular components, of which many have been shown to potentially contribute to neurodegeneration [66], the molecular underpinnings of these findings in relation to the development of AD remain poorly understood. Results from studies of the AD mouse model Tg2576 crossed onto an *SNCA* knockout background proposed that loss of αSyn increased the Aβ plaque load in all forebrain areas by the age of 18 months [23]. In support, it was found that αSyn inhibited amyloid plaque formation in *APPPS1* mice co-expressing the *SNCA* gene. The authors of the same study further demonstrated that the seeding activity of injected Aβ containing brain homogenates was reduced, and that Aβ deposition was suppressed in grafted tissue from the [A30P]aSYN transgenic mouse model [2]. The mechanisms underlying the inverse association between Aβ plaque formation and αSyn have yet to be determined, and may be complicated by the existence of various αSyn species (monomers, oligomers, fibrils) which individually may have differing effects on AD pathophysiology. For example, Larson et al. recently reported that increasing oligomeric αSyn selectively decreased presynaptic proteins and cognitive performance in the bigenic J20xTgI2.2 AD mouse model where both monomeric and oligomeric αSyn increase with age, [27].

In addition to the overlapping neuropathological features between AD and synucleinopathies like PD and DLB, but not multiple system atrophy [42], these disorders also share the *APOEε4* allele as a strong genetic risk factor. The *APOEε4* allele increases the risk of AD by up to 15-fold and the risk of DLB by up to 6-fold [7, 10]. Furthermore, the *APOEε4* allele also increases the risk of PD and decreases the age of disease onset [30]. The exact molecular pathways underlying the increased risk of several neurodegenerative disorders in *APOEε4*-carriers have yet to be identified but the urgency to do so is clearly illustrated by the rather high frequency (~ 14%) of this allele in the normal population [32].

In the current study we aimed to assess the pathophysiological relevance of αSyn levels in CSF to the development of AD, including surrogate markers of AD neuropathology, in a clinical setting. We further aimed to investigate potential effects of the *APOE*ε4 risk allele on identified relationships. For our purposes we used two cohorts, a longitudinally followed cohort of patients with sporadic MCI and AD to investigate potential relationships between αSyn and disease progression. The second cohort was comprised of a cross-sectional sample from the Dominantly Inherited Alzheimer’s Disease Network (DIAN) including participants with autosomal dominant mutations in the *APP*, *PSEN1* and *PSEN2* genes causing early-onset autosomal dominant Alzheimer’s disease (ADAD), and their non-mutation carrying relative control participants [3]. The latter cohort was included to enable specific analyses of relationships between αSyn and the development of symptoms in subjects that will de facto develop Alzheimer’s disease. Furthermore, inclusion of subjects with ADAD mutations also enabled us to assess potential associations between CSF αSyn levels and specific mutations in the genes encoding key components in the Aβ peptide production pathway i.e. the amyloid precursor protein (APP) and the presenilins 1 and 2 (PS1 and PS2).

## Materials and methods

### Study participants

Subjects from two different cohorts were included in the present study. The longitudinally followed cohort totaling *n* = 136 subjects, including *n* = 52 subjects that were cognitively healthy controls and *n* = 84 patients who at baseline were diagnosed with sporadic MCI (*n* = 57) or AD (*n* = 27). Clinical diagnoses of MCI or AD were based on the International Working Group on Mild Cognitive Impairment Criteria [63] and the National Institute of Neurological and Communicative Disorders and Stroke and the Alzheimer’s Disease and Related Disorders Association criteria [34, 35]. The clinical workup and results from parts of this cohort were previously reported [5, 6, 45]. Briefly, consenting participants were recruited by the Department of Neurology at Trondheim University Hospital in Trondheim, Norway on the conditions that individuals had adequate vision and hearing, did not exhibit high alcohol consumption or use of anti-coagulation medication, and had no psychiatric or malignant disease. Cognitively healthy control subjects were recruited from elderly caregivers who were not genetically related to the patients or from societies for retired people in central Norway. Cerebrospinal fluid was drawn at baseline and at intervals of 12-months follow-up examinations over two years. Control individuals had their CSF sampled at baseline only, due to restrictions pertaining to ethical permits; however, they were clinically assessed both at baseline and at the study-end. Cerebrospinal fluid sampling and CSF AD biomarker analysis (for research purposes only) including quantification of Aβ_1–40_, Aβ_1–42_, total tau (t-tau) and tau phosphorylated at Thr181 (p-tau) levels were previously described [6]. Both patient and control groups were *APOE* genotyped from blood samples and underwent cerebral volumetric 3-Tesla MRI (3T-MRI) brain imaging at baseline and after two years. Upon completion of the study, MCI patients were re-classified into two groups based on their baseline and two-year follow-up diagnoses. Patients diagnosed with MCI at baseline and who remained diagnosed with MCI after two years were classified as MCI-MCI (*n* = 30), while patients who were MCI at baseline but that fulfilled the clinical criteria for AD after two years were classified as MCI-AD (*n* = 27). The studies performed on this cohort were approved by the regional ethics committee in Trondheim, Norway (2010/226) and Stockholm, Sweden (2016/771–31/4) and carried out in agreement with the Helsinki Declaration.

Participants in the second cohort were a cross-sectional sample from the multi-site Dominantly Inherited Alzheimer’s Network (DIAN) (https://dian.wustl.edu/our-research/observational-study/). The DIAN registry includes mutation carrying and non-mutation carrying adult biological children from families in which one parent carries an ADAD causative mutation in the *APP*, *PSEN1* or *PSEN2* genes. At inclusion, and during subsequent visits, DIAN participants were assessed neuropsychologically, biochemically (CSF and plasma), and underwent structural neuroimaging using 3T-MRI, and functional neuroimaging using PET combined with either fluorodeoxyglucose (FDG-PET) or PiB to quantify brain glucose metabolism and Aβ deposition respectively [4]. Updated datasets are generated biannually in what are termed dataset “freezes”. For the current study we examined subjects from the DIAN dataset freeze-10 including a total of *n* = 92 ADAD mutation carriers: *n* = 24 *APP*, *n* = 50 *PSEN1*, and *n* = 18 *PSEN2* mutation carriers, as well as *n* = 50 non-mutation carriers who were genetically related to the mutation carriers but did not harbor ADAD mutations. Included subjects were considered as symptomatic if fulfilling the criteria for a Clinical Dementia Rating (CDR) score ≥ 0.5. The purposes of the current study involving DIAN participants were approved by the local ethics committee in Stockholm, Sweden (2016/2114–31/4) and the study was carried out in agreement with the Helsinki Declaration.

### αSyn quantification

Quantification of CSF αSyn in samples from both cohorts was performed using a commercially available sandwich enzyme-linked immunosorbent assay (ELISA) (AnaSpec; California, USA) according to the manufacturer’s instructions and using overnight incubation at 4 °C for the sample incubation step. Samples were assayed alongside freshly prepared standard curves for each individual assay. Standards, study subject and internal control samples were run in duplicates and averaged. Spike recovery experiments were performed to assess matrix effects by adding known concentrations of the supplier-provided αSyn standard to diluted CSF resulting in a spike recovery range of 99–122%. Intra- and inter-assay coefficients of variation were determined by repeated analysis of pooled CSF samples as internal controls resulting in coefficients of variation of < 15% respectively for the two lots of assays used to quantify αSyn in the two cohorts.

### DIAN participant MRI image acquisition

Structural MRI acquisition was performed using the Alzheimer Disease Neuroimaging Initiative (ADNI) protocol [21, 22]. Participating sites were required to pass initial and regular follow-up quality control assessments to insure acquisition conformity. Each participant received an accelerated 3D sagittal T1-weighted MPRAGE on a 3T scanner. A high quality, whole-brain image with 1.1 × 1.1 × 1.2 mm voxels was acquired in approximately 5–6 min. Before further image processing, images were screened for artifacts and protocol compliance by the ADNI imaging core.

### DIAN participant PET image acquisition and processing

A subset of *n* = 132 ADAD participants (mutation carriers *n* = 85, non-mutation carriers *n* = 47) underwent PiB-PET imaging. Each site underwent an initial evaluation to ensure compliance with common PiB-PET ADNI protocols. Amyloid imaging was performed with a bolus injection of ∼15 mCi of PiB. Dynamic acquisition consisted of either a 70-min scan starting at injection or a 30-min scan beginning 40 min post injection. For analysis, the PiB-PET data within the common time frame between 40 and 70 min was used. The ADNI PET Core verified that all PET images were acquired using the established protocol and substantially free of artifacts.

Each subject’s PET data were motion-corrected and registered to their MRI using methods described in detail elsewhere [11, 49]. For each participant, the T1-weighted MRI image was segmented into grey and white matter tissue maps. An inclusive binary gray matter mask was subsequently applied to the resulting atlas to obtain individual gray matter atlases. An automated quantitative image analysis approach was applied using regions of interest generated with FreeSurfer [14] using an in-house software previously described [36, 40, 54]. A regional spread function based approach for partial volume correction of PET data was subsequently implemented using FreeSurfer regions [48] to generate partial volume corrected regional PET data. Partial volume corrected PiB data were used to quantify PiB retention, as it was demonstrated to produce accurate results [53].

Regional partial volume corrected PiB retention data was evaluated in all FreeSurfer regions of interest in Standardized Uptake Value Ratio (SUVR) units, using the brainstem as reference, as this was reported as optimum quantification procedure for PiB-PET in DIAN studies [53]. Overall brain PiB retention was given by the mean cortical PiB retention, calculated as the average PiB SUVR across four cortical regions (prefrontal, gyrus rectus, lateral temporal, and precuneus) as previously used [44]. In addition, exploratory analyses were performed across forty-two bilateral regions of interest from the FreeSurfer atlas. PiB positivity was defined by a mean cortical SUVR uptake using partial volume corrected data and the brainstem as reference region ≥0.72 cutoff value, equivalent to an SUVR of ≥1.42 if using the cerebellar grey matter, as previously described [56].

### Statistics

Normal distribution of the data was assessed by use of the Shapiro-Wilk test. Accordingly, statistical tests applied for cross-sectional group comparisons were either non-parametric (Kruskal-Wallis tests followed by pairwise comparisons with the Mann-Whitney *U* test) or parametric (analysis of variance [ANOVA] or analysis of covariance [ANCOVA] with age entered as covariate when relevant, followed by pairwise comparisons when appropriate, performed with the Student’s *t*-test). The Bonferroni correction was applied to account for multiple comparisons.

In the longitudinal cohort, CSF αSyn levels did not follow a normal distribution and were therefore log-transformed to achieve normal distribution of the data for which parametric tests were used. Longitudinal changes were assessed using the repeated measures multivariate analysis of variance (MANOVA) approach, and correlation analyses were performed by use of the Spearman’s rank correlation test on non-log-transformed data. Data is presented as mean ± standard deviation or as median with range (min-max). A *p*-value of ≤0.05 was considered statistically significant.

In order to compare CSF αSyn levels between subjects with AD pathological versus non-pathological CSF AD biomarker levels receiver operating characteristic (ROC) curve analyses were performed for the longitudinal cohort using the clinical status (cognitively healthy controls versus a baseline AD diagnosis) as the dichotomous variable to establish biochemical cutoff values. Cutoff values were determined based on the highest Youden index defined as *sensitivity + specificity − 1*.

In the DIAN cohort, CSF αSyn levels did not follow a normal distribution hence we log-transformed the data so that the association between αSyn with estimated years from symptom onset (EYO) was evaluated using the Pearson’s correlation test. For brain PiB-PET imaging-related CSF αSyn analyses, linear regression was applied to predict regional PiB-retention as a function of CSF αSyn and EYO as independent variables, separately in ADAD mutation carriers and non-mutation carriers. The same linear regression model was applied after mutation carriers were stratified into asymptomatic (CDR < 0.5) and symptomatic (CDR ≥ 0.5) mutation carriers, as well as for the subset of PiB-positive asymptomatic mutation carriers. Additionally, in mutation carriers the interaction of αSyn and *APOEε4* status (positive/negative) was investigated to elucidate whether the relationship of regional PiB retention and αSyn is influenced by *APOEε4* status. Finally, regional PiB retention was modeled as a function of αSyn and EYO separately in *APOEε4*-positive and *APOEε4-*negative mutation carriers.

All statistical analyses concerning CSF αSyn levels and their association to parameters unrelated to brain imaging were performed in JMP software v.12.1.0 (SAS Institute Inc.). Statistical analyses regarding associations between CSF αSyn and amyloid PET data in the DIAN cohort were performed using the R v.3.1.0 software (The R Foundation for Statistical Computing, http://www.r-project.org/). Graphical representations of significant linear associations were obtained with the ggplot2 package v.1.0.1, as implemented in R. The significance level for all statistical tests and models was set at *p* ≤ 0.05. On the brain volumes, display of regression standardized β coefficients of the significant associations between CSF αSyn and regional PiB-PET were performed using BrainNet viewer [67] (http://www.nitrc.org/projects/bnv/) implemented in MATLAB.

## Results

### Descriptive statistics of the longitudinally followed cohort of sporadic patients

The demographics and clinical characteristics of the longitudinal cohort were in part previously published [45] and are shown in Table 1. The control group was significantly older than the MCI and AD patient groups, thus age was added as a covariate when including control subjects in the statistical analyses. At the two-year follow-up 48% of the subjects diagnosed with MCI at baseline had progressed to an AD diagnosis (MCI-AD), whereas 52% remained stable (MCI-MCI). The patient dropout rate at 24-months was 17% of the MCI-MCI patients, 7% of the MCI-AD patients, and 15% of the AD patients. As expected, the Mini-Mental State Examination (MMSE) scores differed significantly amongst the investigated groups with AD patients displaying the lowest scores (*p* < 0.001). In comparison to controls all individual patient groups exhibited altered levels of CSF AD biomarkers including Aβ_1–42_, Aβ_1–40_, Aβ_42/40_, t-tau and p-tau. A much lower frequency of *APOEε4* carriers was observed in the control group (38%) compared to the MCI (66%) and AD patient groups (82%) (*p* < 0.001).

**Table 1:** Longitudinal cohort baseline characteristics

	Controls	MCI			Alzheimer’s disease	p-value^c^
		All	MCI-MCI	MCI-AD		
N	57	54	27	27	27	
Sex (% f/m)	65/35	53/47	44/56	59/41	52/48	- ^d^
Age at examination (yrs)^a^	68 ± 5	65 ± 6	66 ± 6	64 ± 4	64 ± 6	** ^e^
MMSE^b^	30 (28–30)	28 (23–30)	28 (25–30)	27 (23–29)	23 (16–27)	*** ^f^
*APOEε4* carrier (%)	38 (*n* = 55)	66	63	74	81	*** ^d^
CSF	969.7	546.5	574.3	539.0	476.8	*** ^f^
Aβ_1–42_	(500–1674)	(173–1269)	(173–1269)	(283–1060)	(212–1092)	
(pg/mL)^b^	(*n* = 45)					
CSF	17.1	13.5	12.9	14.2	14.9	** ^f^
Aβ_1–40_	(11–41)	(4–31)	(4–31)	(8–23)	(7–29)	
(ng/mL) ^b^	(*n* = 41)		(*n* = 26)	(*n* = 23)	(*n* = 26)	
CSF	0.059	0.042	0.049	0.039	0.034	*** ^f^
Aβ_42/40_^b^	(0.013–0.096)	(0.008–0.118)	(0.008–0.118)	(0.012–0.088)	(0.007–0.080)	
	(*n* = 41)		(*n* = 26)	(*n* = 23)	(*n* = 26)	
CSF	269.0	447.9	315.2	550.8	624.2	*** ^f^
t-tau	(138–1314)	(99–2325)	(99–1057)	(163–2325)	(177–1540)	
(pg/mL)^b^	(*n* = 46)					
CSF	53.5	69.9	53.0	85.6	90.8	*** ^f^
p-tau	(33–135)	(16–169)	(16–131)	(37–169)	(28–157)	
(pg/mL)^b^	(*n* = 46)					

*MCI* = mild cognitive impairment MCI-MCI = MCI patients who remained MCI at the 24-month follow up

MCI-AD = MCI patients who converted to Alzheimer’s disease at the 24-month follow up

MMSE = Mini-Mental State Examination score

a = mean ± standard deviation b = median (minimum-maximum)

c = *P*-value reflecting potential differences between the groups Controls, mild cognitive impairment and Alzheimer’s disease d = χ^2^ test e = one-way ANOVA f = Kruskal-Wallis test - = non-significant

** = *p* ≤ 0.01 *** = *p* ≤ 0.001

### Higher baseline CSF αSyn levels in MCI patients converting to sporadic AD

**Fig. 1:**
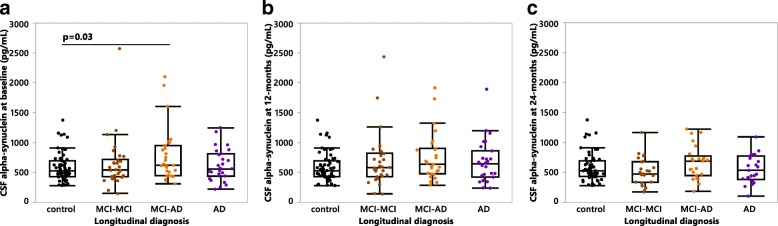
CSF αSyn levels in sporadic MCI and Alzheimer’s disease cohort. Quantification of CSF αSyn levels at (**a**) baseline, (**b**) 12-months and (**c**) 24-months in the four longitudinally diagnosed patient groups: control = cognitively healthy controls, MCI-MCI = MCI patients who remained MCI at the 24-month follow up, MCI-AD = MCI patients who converted to Alzheimer’s disease at the 24-month follow up, AD = patients diagnosed with AD at baseline. *P*-values were calculated using a one-way ANCOVA of log-transformed data with age entered as a covariate with post-hoc testing by use of the student’s *t*-test. Bonferroni correction was used to account for multiple comparisons (a: *n* = 6 comparisons, b-c: *n* = 3 comparisons). Results are displayed without log-transformation or age-correction (raw data)

We first sought to compare CSF αSyn levels between the diagnostic groups. At baseline, MCI patients that after 24 months fulfilled the criteria for an AD diagnosis (MCI-AD) exhibited the highest CSF αSyn levels compared to controls (Fig. 1a), while CSF αSyn levels did not significantly differ between controls and the non-converting MCI patients (MCI-MCI), and the AD group (Fig. 1a). No differences in CSF αSyn levels were noted between the patient groups at either the 12-month (Fig. 1b) or 24-month (Fig. 1c) follow-ups.

### Association between *APOEε4* and higher CSF αSyn levels in sporadic AD patients

**Fig. 2:**
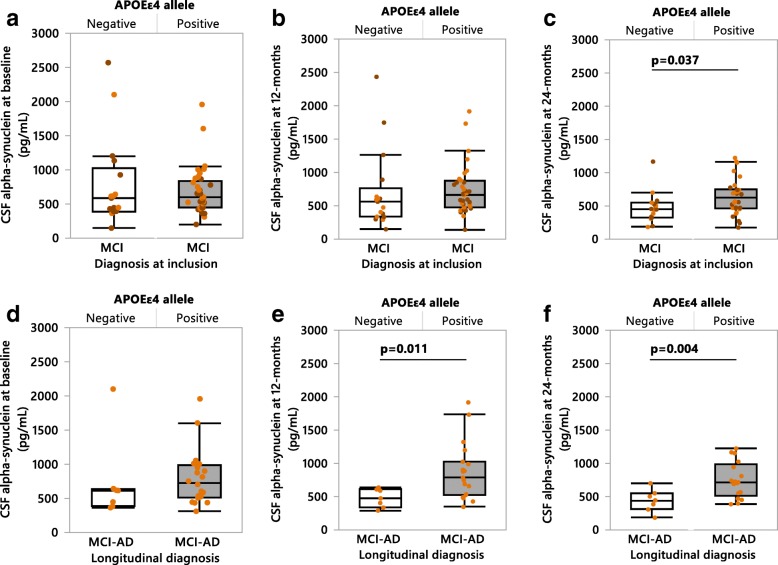
CSF αSyn measured in *APOEε4*-positive versus *APOEε4*-negative sporadic MCI patients. **a**-**c** CSF αSyn quantified in MCI patients at baseline, 12- and 24-months respectively. *APOEε4*-positive patients are shown as grey shaded boxes, *APOEε4*-negative patients are white boxes. Orange data points represent patients longitudinally diagnosed as MCI-AD, brown points represent MCI-MCI patients. **d-f** CSF αSyn quantified in MCI-AD diagnosed patients at baseline, 12- and 24-months respectively. *P*-values were calculated using the one-way ANCOVA of log-transformed data, with age entered as a covariate with post-hoc testing by use of the student’s *t*-test. Results are displayed without log-transformation or age-correction (raw data). MCI-AD = MCI patients who converted to Alzheimer’s disease at the 24-month follow up

**Fig. 3:**
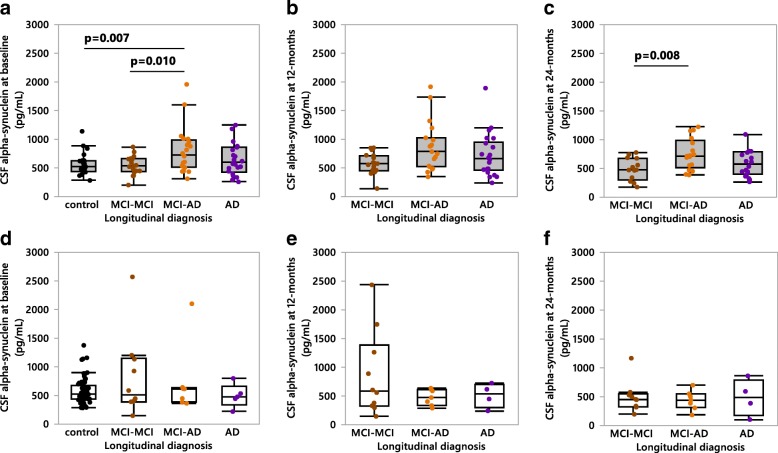
CSF αSyn measured in the subjects of the longitudinal cohort based on *APOE*ε4 status. **a-c** *APOEε4*-positive (grey boxes) subjects examined at baseline, 12- and 24-months respectively. **d-f** *APOEε4*-negative patients examined at baseline, 12- and 24-months respectively. *P*-values were calculated using the one-way ANCOVA of log-transformed data, with age entered as a covariate with post-hoc testing by use of the student’s *t*-test. Bonferroni correction was used to account for multiple comparisons (a: n = 6 comparisons, b-f: *n* = 3 comparisons). Results are displayed without log-transformation or age-correction (raw data). MCI-MCI = MCI patients who remained MCI at the 24-month follow up, MCI-AD = MCI patients who converted to Alzheimer’s disease at the 24-month follow up, AD = patients diagnosed with Alzheimer’s disease at baseline

**Fig. 4:**
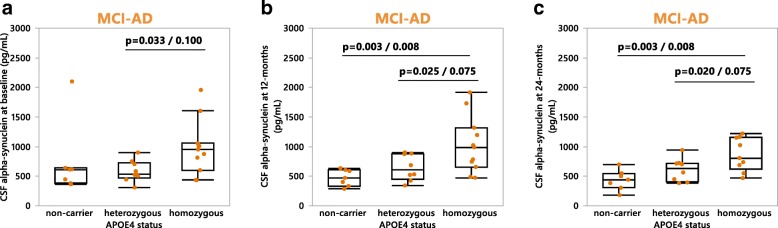
Cerebrospinal fluid αSyn levels in MCI-AD patients at: **a** baseline (non-carrier *n* = 7, heterozygous *n* = 9, homozygous *n* = 11), **b** 12-months (non-carrier *n* = 7, heterozygous *n* = 8, homozygous *n* = 11), and **c** 24-months (non-carrier *n* = 7, heterozygous *n* = 8, homozygous *n* = 9). *P*-values are presented first as uncorrected followed by Bonferroni corrected (a-c: n = 3 comparisons). In **a** *P*-values were calculated using the non-parametric Kruskal-Wallis with post-hoc testing by use of the Mann-Whitney *U* test. Bonferroni correction was used to account for multiple comparisons. In (**b-c**) *P*-values were calculated using the one-way ANCOVA with age entered as a covariate with post-hoc testing by use of the student’s *t*-test. Results are displayed without age-correction (raw data). MCI-AD = MCI patients who converted to Alzheimer’s disease at the 24-month follow up

As *APOEε4* is a common genetic risk factor for both synucleinopathies and AD [7, 10, 30], we assessed the effect of *APOEε4* on CSF αSyn levels. Within the MCI patient group *APOEε4* carriers exhibited higher αSyn levels compared to non-carriers at the 24-month follow up (Fig. 2c). Additionally, MCI-AD *APOEε4* carriers exhibited higher CSF αSyn than non-carriers at the 12-month (Fig. 2e) and 24-month (Fig. 2f) follow-ups. The MCI-AD *APOEε4* carrying patient group also exhibited significantly higher CSF αSyn levels at baseline compared to *APOEε4* carrying MCI-MCI patients and controls (Fig. 3a), while no differences in CSF αSyn were found at the 12-month follow up (Fig. 3b). However, at 24-months MCI-AD *APOEε4* carriers again had higher CSF αSyn than MCI-MCI *APOEε4* carriers (Fig. 3c). This difference in CSF αSyn levels was absent when comparing *APOEε4* non-carrier patient groups at any time point (Fig. 3d-f). Intriguingly, the elevated CSF αSyn levels found in MCI-AD patients exhibited a dose-dependent relationship with the *APOEε4* allele at all three time points (Fig. 4a-c).

**Fig. 5:**
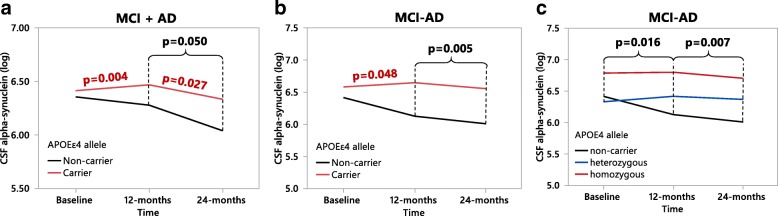
Longitudinal assessment of mean CSF αSyn values at baseline, 12-months and 24-months in (**a)** a pooled MCI and Alzheimer’s disease (AD) patient group classified as either *APOEε4* carriers (baseline to 12-months *n* = 58, 12-months to 24-months *n* = 47) or non-carriers (baseline to 12-months *n* = 21, 12-months to 24-months *n* = 19). **b** the MCI-AD patient group classified as either *APOEε4* carriers (baseline to 12-months *n* = 19, 12-months to 24-months *n* = 16) or non-carriers (baseline to 12-months *n* = 7, 12-months to 24-months *n* = 7), **c** the MCI-AD patient group classified as either *APOEε4* non-carriers (baseline to 12-months *n* = 7, 12-months to 24-months *n* = 7), heterozygotes (baseline to 12-months *n* = 8, 12-months to 24-months *n* = 7) or homozygotes (baseline to 12-months *n* = 11, 12-months to 24-months *n* = 9). *P*-values within patient groups over 1-year intervals are shown in red along the trend lines, and between-group *p*-values are indicated in black. *P*-values were calculated using repeated measures MANOVA analysis of log-transformed data. MCI-AD = MCI patients who converted to Alzheimer’s disease at the 24-month follow up

To assess any longitudinal changes in CSF αSyn levels occurring within- or between the patient groups we performed repeated measures MANOVAs spanning the intervals of: baseline to 12-months, baseline to 24-months, and 12-months to 24-months (Fig. 5). When all patients (diagnosed with MCI or AD at baseline) were grouped as either *APOEε4* carriers or non-carriers, *APOEε4* carriers showed increasing CSF αSyn levels (+ 17%) from baseline to 12-months, followed by a decrease (− 10%) from 12- to 24-months (Fig. 5a). Patients that did not carry the *APOEε4* variant consistently exhibited a longitudinal decline in CSF αSyn levels which reached statistical significance between the 12- and 24-months examinations (Fig. 5a), thus proposing potentially different pathological processes for *APOEε4* carriers versus non-carriers. These findings prompted us to also subdivide the MCI-AD patient group into *APOEε4* carriers or non-carriers. We found a significant increase in CSF αSyn (+ 5%) in MCI-AD *APOEε4* carriers from baseline to 12-months, while also observing elevated CSF αSyn in MCI-AD *APOEε4* carriers versus non-carriers from 12- to 24-months (Fig. 5b). Moreover, when the MCI-AD patient group was further subdivided by *APOEε4* zygosity we found significant differences in CSF αSyn levels from baseline to 12-months, and 12- to 24-months (Fig. 5c). A significant difference between *APOEε4* homozygotes, heterozygotes, and non-carriers was also found in the AD group between 12-months and 24-months (data not shown).

### Links between CSF αSyn, AD biomarkers and cognition in sporadic patients

In the absence of established brain imaging tools to assess ante-mortem αSyn burden in the brain, we assessed potential correlations between CSF αSyn levels and established fluid surrogate markers of AD pathology and global cognitive test scores. At baseline, 12-months, and 24-months CSF αSyn showed a positive correlation to CSF Aβ_1–40_, t-tau, and p-tau in all patient groups and controls (Table 2). All MCI patients showed a marked strengthening in the correlations of CSF αSyn to CSF Aβ_1–40_, t-tau, and p-tau between baseline and 12-months, and were the strongest in the MCI-AD group compared to all other groups at 12-months (Table 2). In MCI-MCI patients the correlations of CSF αSyn to CSF Aβ_1–40_ and p-tau strengthened from baseline to 24-months, while the MCI-AD group exhibited the strongest correlation between CSF αSyn and t-tau (all above Spearman’s *ρ* = 0.9 respectively) (Table 2). The AD group exhibited a modest increase in the correlations between CSF αSyn and CSF t-tau and p-tau between baseline and 12-months, but showed weakening of the correlation between CSF αSyn and Aβ_1–40_ over the same time interval. Additionally, at 24-months the AD group was the only group of patients at any time point to exhibit a significant correlation between CSF αSyn and Aβ_1–42_. At 24-months however, the AD group exhibited the weakest correlations between CSF αSyn and the CSF AD biomarker panel. Intriguingly, our analyses revealed a significant association between CSF αSyn levels and cognition, in which higher CSF αSyn levels were significantly correlated to lower MMSE scores in the MCI-AD group at baseline (Table 2); moreover, the correlation was solely attributable to *APOEε4* carrying MCI-AD patients (*ρ*=-0.607, *p*=0.005) (non-carrier *ρ*=-0.618, *p*=0.140).

**Table 2:** Longitudinal cohort CSF αSyn correlation analyses^ǂ^

Time	Longitudinal diagnosis	MMSE	CSF Aβ_1-42_	CSF Aβ_1-40_	CSF Aβ_42/40_	CSF t-tau	CSF p-tau
Baseline	Control (*n*=57)	-	- (*n*=41)	0.616*** (*n*=37)	-0.542*** (*n*=37)	0.871*** (*n*=42)	0.766*** (*n*=42)
	MCI-MCI (*n*=27)	-	-	0.472** (*n*=26)	- (*n*=26)	0.577***	0.651***
	MCI-AD (*n*=27)	-0.605***	-	0.640*** (*n*=23)	-0.457* (*n*=23)	0.736***	0.694***
	AD (*n*=27)	-	-	0.758*** (*n*=26)	- (*n*=26)	0.722***	0.738***
12-months	MCI-MCI (*n*=27)	-	- (*n*=15)	0.703*** (*n*=15)	-0.575* (*n*=15)	0.804*** (*n*=15)	0.849*** (*n*=15)
	MCI-AD (*n*=25)	-	- (*n*=19)	0.863*** (*n*=18)	-0.732*** (*n*=18)	0.913*** (*n*=19)	0.927*** (*n*=19)
	AD (*n*=24)	-	- (*n*=18)	0.691*** (*n*=18)	- (*n*=18)	0.804*** (*n*=18)	0.770*** (*n*=18)
24-months	MCI-MCI (*n*=24)	-	- (*n*=14)	0.925*** (*n*=14)	- (*n*=14)	0.782*** (*n*=14)	0.924*** (*n*=14)
	MCI-AD (*n*=24)	-	- (*n*=18)	0.716*** (*n*=17)	-0.556* (*n*=17)	0.902*** (*n*=18)	0.919*** (*n*=18)
	AD (*n*=18)	-	0.662** (*n*=16)	0.585* (*n*=16)	- (*n*=16)	0.741** (*n*=16)	0.650** (*n*=16)

^ǂ^All correlations calculated using the Spearman’s rank correlation test

MCI-MCI= MCI patients who remained MCI at the 24-month follow up

MCI-AD= MCI patients who converted to Alzheimer’s disease at the 24-month follow up

*AD* patients diagnosed with Alzheimer’s disease at baseline, *MMSE* Mini-Mental State Examination score

*p ≤0.05, **p ≤0.01, ***p ≤0.001

### αSyn levels in sporadic patients with or without pathological CSF AD biomarkers

**Fig. 6:**
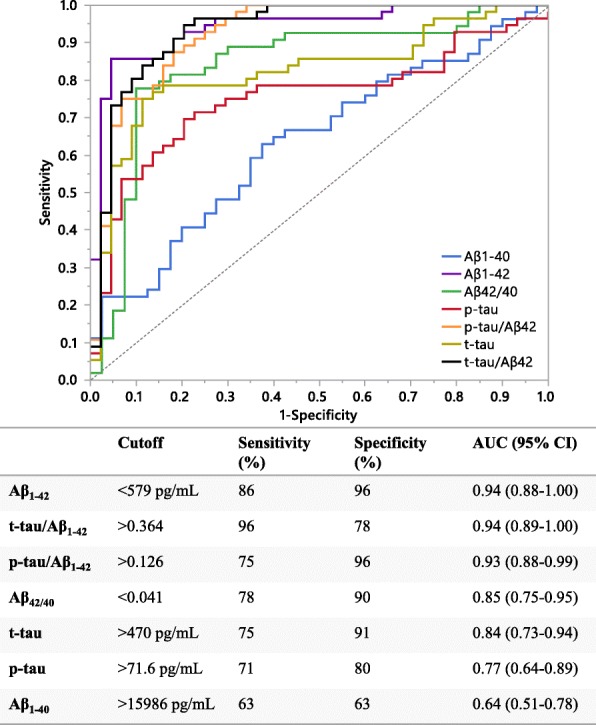
Receiver operator characteristic (ROC) curves of AD CSF biomarkers. For each CSF biomarker analyte or ratio the table indicates the cutoff value, sensitivity (%), specificity (%), and area under the ROC curve (AUC) with the corresponding 95% confidence interval. A clinical diagnosis of healthy control versus AD was used as the dichotomous variable to define CSF cutoffs based on the best performing Youden index

**Fig. 7:**
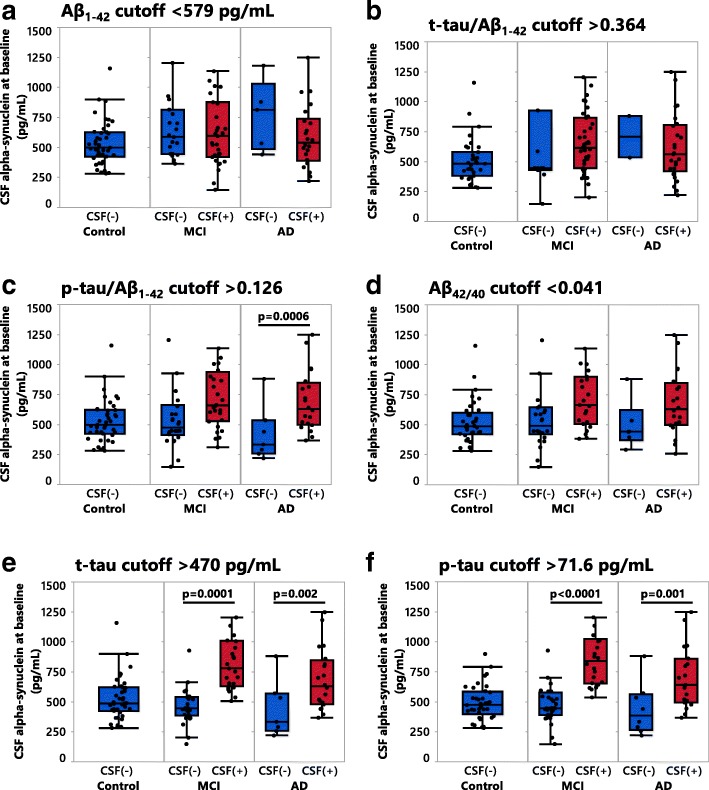
Baseline levels of CSF αSyn in the longitudinal cohort based on ROC cutoff values. **a-f** Clinically diagnosed subjects subdivided into either CSF Alzheimer’s disease (AD) biomarker negative (CSF(−)) or positive (CSF(+)) groups based on cutoff values. *P*-values were calculated using the one-way ANCOVA of log-transformed data, with age entered as a covariate with post-hoc testing by use of the student’s *t*-test. Results are displayed without log-transformation

The MCI and AD patients enrolled in the longitudinal cohort were diagnosed according to clinical guidelines [34, 63] without workup of CSF AD biomarkers to support the clinical diagnosis, however, for research purposes the CSF biomarkers were assessed. With the rationale to compare CSF αSyn levels between patients with pathological versus without pathological CSF AD biomarker levels, we employed ROC analyses to produce cutoffs for the core CSF AD biomarkers Aβ_1–42_, t-tau, p-tau and the conventional ratios thereof [47]. We then stratified the patients into groups with and without pathological CSF AD biomarker levels (Fig. 6). Cutoffs were determined by comparing AD biomarker levels between cognitively healthy controls and AD patients at baseline. The resulting ROC axes were labeled as sensitivity and 1-specificity; the accompanying results table provided the individual cutoff values, sensitivity, specificity, and the area under the curve (AUC) with its respective 95% confidence interval (95% CI) (Fig. 6). The cutoff values (Fig. 6) for each CSF biomarker were then used to compare CSF αSyn levels between CSF AD biomarker negative (CSF(−)) and positive (CSF(+)) subjects within the diagnostic groups (Fig. 7). When utilizing the cutoffs for CSF t-tau (> 470 pg/mL) and p-tau (> 71.6 pg/mL) in the MCI and AD patients, we discovered significant differences where CSF(+) patients had elevated αSyn compared to CSF(−) patients (Fig. 7e-f). Additionally, the CSF p-tau/Aβ_42_ cutoff (> 0.126) applied in the AD patient group showed that the CSF(+) group had higher CSF αSyn than the CSF(−) group (Fig. 7c).

### Descriptive statistics of DIAN participants

In the ADAD mutation carrying participants from the DIAN study, *n* = 6 *APP*, *n* = 33 *PSEN1* and *n* = 3 *PSEN2* mutations were represented (data not shown). Of the *n* = 92 included ADAD mutation carriers 34.8% had cognitive symptoms (CDR ≥0.5). Specifically, 37.5% of *APP*, 42.0% of *PSEN1* and 11.1% of *PSEN2* mutation carriers were symptomatic (Table 3). More than half of the *PSEN2* mutation carriers carried the *APOEε4* allele, whereas a quarter of the *APP* mutation carriers and less than 20% of the *PSEN1* mutation carriers were *APOEε4* carriers (Table 3). In all three ADAD mutation groups, there was a higher percentage of *APOEε4* carriers amongst the symptomatic mutation carriers (*p* < 0.01). Further, we found significant variability in the EYO, MMSE and CDR scores, and CSF levels of Aβ_1–42_, Aβ_42/40_, t-tau, and p-tau between ADAD mutation carriers and non-mutation carriers (Table 3). When the three ADAD mutation carrier groups where subdivided into symptomatic (CDR ≥ 0.5) and asymptomatic (CDR < 0.5) individuals it was found that age of examination, EYO and MMSE scores as well as CSF Aβ_42/40_ and CSF levels of t-tau, and p-tau significantly differed between symptomatic versus asymptomatic in *APP* mutation carriers (Table 3). Age of examination, EYO, MMSE and CDR scores, CSF Aβ_42/40_, Aβ_1–42_, t-tau, and p-tau significantly differed between symptomatic and asymptomatic *PSEN1* mutation carriers, while no differences could be observed in the *PSEN2* mutation carriers due to insufficient sample size (Table 3).

**Table 3:** DIAN cohort baseline characteristics

	Non-Mutation Carriers	All Mutation Carriers	*APP* Mutation Carriers			*PSEN1* Mutation Carriers			*PSEN2* Mutation Carriers			*p*-value^a^
			All	Asym	Sym	All	Asym	Sym	All	Asym	Sym	
N	50	92	24	15	9	50	29	21	18	16	2	
Sex (% f/m)	56/44	55/45	63/37	67/33	56/44	60/40	52/48	71/29	33/66	32/68	50/50	–
Age at examination (yrs)	40 ± 11	40 ± 10	42 ± 11	39 ± 12	49 ± 6	39 ± 10	34 ± 9	46 ± 8	40 ± 10	38 ± 9	57	***^c^
EYO	−9 ± 11	−7 ± 12	5 ± 11	−11 ± 11	4±3	−6 ± 12	−14 ± 10	5 ± 4	−13 ± 11	−15 ± 9	7	–
MMSE^§^	29 ± 1	27 ± 5	27 ± 5	29 ± 1	23 ± 7	26 ± 6	29 ± 1	21 ± 7	29 ± 3	30 ± 1	20	*^c^
CDR	0 ± 0	0.3 ± 0.6	0.3 ± 0.5	0 ± 0	1 ± 0.5	0.4 ± 0.7	0 ± 0	1 ± 0.8	0.1 ± 0.5	0 ± 0	1	-^d^
*APOEε4* carrier (%)	36	27	25	20	33	18	14	24	56	50	100	**^e^
CSF Aβ_1–42_ (pg/mL)	858 ±264	613 ± 320 (*n* = 89)	613 ± 353 (*n* = 23)	734 ± 393 (*n* = 14)	425 ± 164	621 ± 335 (*n* = 49)	770 ± 318 (*n* = 28)	422 ± 243	592 ± 233 (*n* = 17)	596 ± 249 (*n* = 15)	565	-^c^
CSF Aβ_1–40_ (pg/mL)	8173 ± 2751	7477 ± 3146 (*n* = 89)	6639 ± 3278 (*n* = 23)	6302 ± 3494 (*n* = 14)	7162 ± 3033	7563 ± 2785 (*n* = 49)	8313 ± 2918 (*n* = 28)	6563 ± 2300	8363 ± 3810 (*n* = 17)	8811 ± 3776 (*n* = 15)	5005	-^c^
CSF Aβ_42/40_	0.115 ± 0.045	0.092 ± 0.052 (*n* = 89)	0.106 ± 0.065 (*n* = 23)	0.128 ± 0.066 (*n* = 14)	0.072 ± 0.049	0.088 ± 0.048 (*n* = 49)	0.102 ± 0.047 (*n* = 28)	0.070 ± 0.045	0.082 ± 0.037 (*n* = 17)	0.076 ± 0.031 (*n* = 15)	0.129	-^c^
CSF t-tau (pg/mL)	56 ± 26 (*n* = 49)	106 ± 73 (*n* = 87)	90 ± 64 (*n* = 23)	60 ± 30 (*n* = 14)	137 ± 76	124 ± 82 (*n* = 47)	94 ± 56 (*n* = 26)	162 ± 94	75 ± 39 (*n* = 17)	72 ± 40 (*n* = 15)	98	*^c^
CSF p-tau (pg/mL)	29 ± 14 (*n* = 49)	54 ± 34 (*n* = 89)	46 ± 25 (*n* = 23)	32 ± 11 (*n* = 14)	69 ± 24	60 ± 37 (*n* = 49)	45 ± 28 (*n* = 28)	81 ± 38	45 ± 31 (*n* = 17)	48 ± 32 (*n* = 15)	29	-^c^

*Asym* = asymptomatic *Sym* = symptomatic *EYO* = estimated yeats from symptom onset *MMSE* = Mini-Mental State Exam scorre

*CDR* = Clinical Dementia Rating score *a* = *p*-value versus all mutation carriers *c* = Kruskal-Wallis test *d* = Mann-Whitney *U* test e = χ^2^ test § = non-normally distributed data presented at mean ± standard deviation in order to maintain patient anonymity

**-** = non-significant * = *p* ≤ 0.05 ** = *p* ≤ 0.01 *** = *p* ≤ 0.001

### Cerebrospinal fluid αSyn levels in ADAD mutation carriers are related to onset of cognitive symptoms

**Fig. 8:**
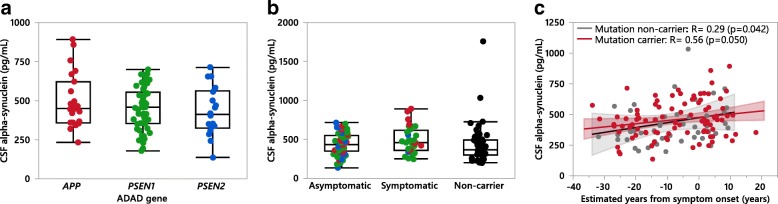
CSF αSyn levels quantified in participants from the DIAN cohort. **a** CSF αSyn levels grouped by ADAD gene mutation. **b** CSF αSyn in ADAD non-mutation carriers versus asymptomatic and symptomatic ADAD mutation carriers. Data point coloring represents the three ADAD genes as shown in (**a**). *P*-values are presented first as uncorrected followed by Bonferroni corrected (a-b: *n* = 3 comparisons). *P*-values calculated using the non-parametric Kruskal-Wallis test and a Bonferroni correction was used to account for multiple comparisons. **c** Correlation between CSF αSyn and the estimated years from symptom onset (EYO) in ADAD mutation carriers and non-mutation carriers; correlations are calculated using Pearson’s correlation test after log-transformation of the CSF αSyn data and results are displayed without log-transformation or age-correction (raw data)

After pooling all individual gene mutations into their respective groups (*APP*, *PSEN1* and *PSEN2* mutation carriers) we compared the CSF αSyn levels between the three groups and found no significant differences between the groups of ADAD mutation carriers or versus non-mutation carriers (Fig. 8a). Upon subdividing the ADAD mutation carriers into either asymptomatic or symptomatic individuals we found that symptomatic mutation carriers had higher CSF αSyn compared to non-mutation carriers (Fig. 8b); no further differences were found when *APOEε4* status was included as a covariate (data not shown). Importantly, the temporal trajectory of CSF αSyn versus EYO showed a weak-to-moderate increase in both ADAD mutation carriers (Pearson’s *R* = 0.20; *p* = 0.056) and in non-carriers (Pearson’s *R* = 0.31; *p* = 0.032), where the confidence bands around both trajectories showed a substantial overlap (Fig. 8c).

### Cerebrospinal fluid αSyn and AD biomarker associations in the DIAN cohort

To assess links between CSF αSyn levels and AD brain pathology using surrogate CSF AD biomarkers in the DIAN participants we investigated potential correlations between CSF αSyn and CSF levels of Aβ_1–42_, Aβ_1–40_, Aβ_42/40_, t-tau, and p-tau in ADAD non-mutation carriers and the three ADAD mutation carrier groups sorted into asymptomatic and symptomatic subgroups (the *n* = 2 *PSEN1* symptomatic patient group was excluded from analysis) (Table 4). We found no correlations between CSF αSyn and Aβ_1–42_ when combining asymptomatic and symptomatic ADAD mutation carriers, but did find a positive correlation between CSF αSyn and Aβ_1–40_ in non-mutation carriers, and both asymptomatic and symptomatic *APP* and *PSEN1* mutation carriers. Inverse correlations between CSF αSyn and Aβ_42/40_ were found in symptomatic *APP* and *PSEN1* mutation carriers, but most notably in the symptomatic *APP* mutation carrier group (Table 4). There were strong positive associations between CSF αSyn and t-tau in ADAD non-mutation carriers and in all three mutation carrier groups, both asymptomatic and symptomatic, with the strongest correlations found in asymptomatic *PSEN1* and symptomatic *APP* mutation carriers (Table 4). Asymptomatic *PSEN1* mutation carriers also exhibited a significant positive relationship between CSF αSyn and p-tau while both non-mutation carriers and *PSEN2* mutation carriers demonstrated a significant positive correlation between CSF αSyn and p-tau levels.

**Table 4:** DIAN cohort CSF αSyn correlation analyses^a^

	Non- mutation carriers	*APP* mutation carriers			*PSEN1* mutation carriers		*PSEN2* mutation carriers	
		Asym (*n* = 15)	Sym (*n* = 9)		Asym (*n* = 29)	Sym (*n* = 21)	Asym (*n* = 16)	Sym (*n* = 2)
CSF Aβ_1–42_	0.595***	–	–		–	–	–	¤
CSF Aβ_1–40_	0.744***	0.610*	0.717*		0.526**	0.788***	–	¤
CSF Aβ_42/40_	–	–	−0.800**		–	−0.464*	–	¤
CSF t-tau	0.716***	0.676*	0.917***		0.943***	0.664***	0.736**	¤
CSF p-tau	0.544***	–	–		0.809***	0.662***	–	¤

^a^ = All correlations calculated using the Spearman’s rank correlation test

*Asym* = asymptomatic Sym = symptomatic

¤ = not determined * = *p* ≤ 0.05 ** = *p* ≤ 0.01 *** = *p* ≤ 0.001

### Associations between brain Aβ plaque load and CSF αSyn in the DIAN cohort

Data from PiB-PET examinations reflecting in vivo amyloid plaque burden were available for *n* = 132 of the DIAN participants (dataset freeze 10). The frequency of PiB-positive ((SUVR/brainstem) ≥0.72) subjects was; 49.1% (28/57) of asymptomatic mutation carriers, 89.3% (25/28) of symptomatic mutation carriers, and 4.3% (2/47) of non-mutation carriers. As expected, PiB-retention increased with EYO in mutation carriers, while no significant increase was noted in non-mutation carriers (data not shown).

**Fig. 9:**
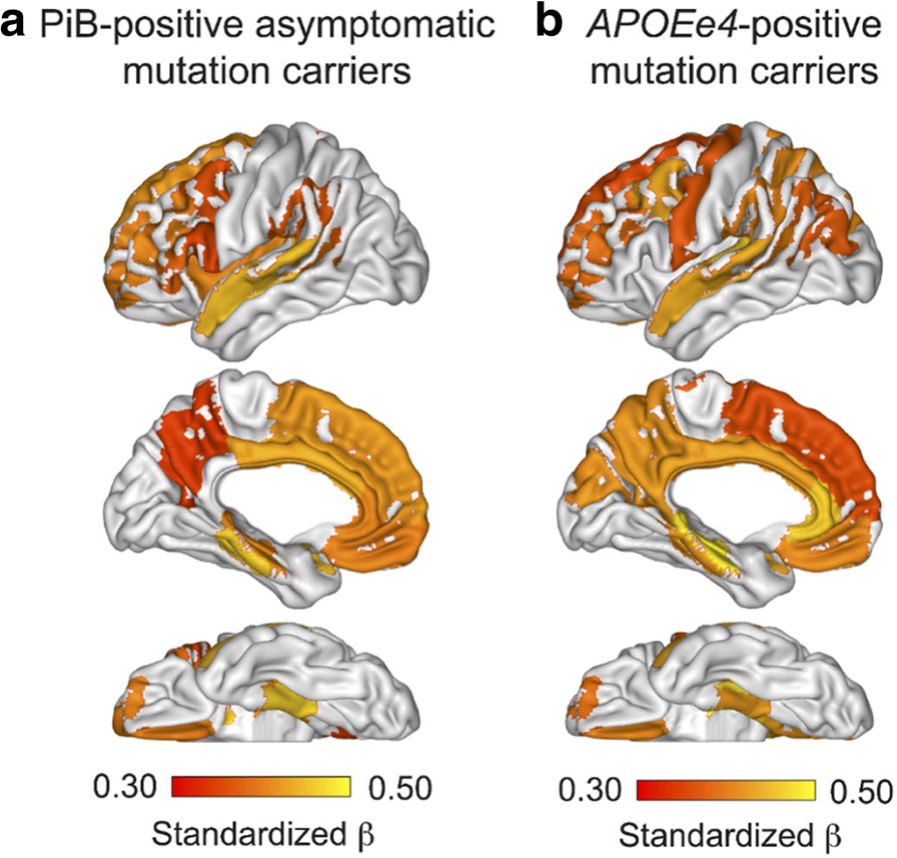
Brain maps depicting significant associations between CSF αSyn and PiB-retention in ADAD mutation carrier subgroups. **a** PiB-positive asymptomatic (CDR < 0.5), **b** *APOEε4*-positive (CDR < 0.5). The significant associations between CSF αSyn and PiB are represented by the standardized β coefficient corresponding to independent predictor αSyn in the linear regression model for PiB ~ αSyn + EYO, where EYO is estimated years from symptom onset. Increasing positive associations (increasing β values) are labeled from red to yellow

Potential associations between CSF αSyn levels and in vivo amyloid plaque burden in subjects from the DIAN cohort were investigated by analyzing the linear regressions of mean cortical PiB-retention versus CSF αSyn in ADAD mutation carriers and non-mutation carriers separately, as well as stratifying mutation carriers into asymptomatic and symptomatic groups, adding EYO as independent predictor in all regression models; these four models resulted in no significant associations between mean cortical PiB and αSyn (Table 5). When looking at the subset of PiB-positive asymptomatic ADAD mutation carriers, however, the same linear regression model showed a significant positive association between mean cortical PiB and αSyn (β = 0.44, *p* = 0.010); results from exploratory linear regression models in 42 regions of interest in PiB-positive asymptomatic mutation carriers are illustrated in Fig. 9a in which the regional regression β coefficients are displayed on a 3D brain surface. The strongest associations between CSF αSyn and amyloid plaque load in PiB-positive asymptomatic ADAD mutation carriers were found in regions including the posterior cingulate, superior temporal and frontal (Fig. 9a).

**Table 5:** Linear regression models relating mean cortical ^11^C-Pittsburgh Compound-B uptake and αSyn in ADAD mutation carriers

Model (PiB-positive asymptomatic mutation carriers): Mean cortical PiB ~ αSyn + EYO				
Independent predictors	Standardized β	Std. Error	*t* value	*p* value
αSyn	0.375	0.00047	2.348	0.027
EYO	0.444	0.00676	2.780	0.010
Model (all mutation carriers): Mean cortical PiB ~ αSyn + *APOEε4* + EYO + αSyn*APOEε4				
Independent predictors	Estimate	Std. Error	*t* value	*p* value
αSyn	−0.00038	0.00039	−0.970	0.335
*APOEε4*	−0.98698	0.44767	−2.205	0.030
EYO	0.02682	0.00435	6.164	0.000
αSyn*APOEε4	0.00244	0.00099	2.468	0.016
Model (*APOEε4*-positive mutation carriers): Mean cortical PiB ~ αSyn + EYO				
Independent predictors	Standardized β	Std. Error	*t* value	*p* value
αSyn	0.393	0.00088	2.321	0.032
EYO	0.533	0.00864	3.147	0.005

PiB = ^11^C-Pittsburgh Compound-B *EYO* = estimated years from symptom onset

A potential effect of *APOEε4* on the association between PiB and αSyn in ADAD mutation carriers was assessed by the linear regression model: Mean cortical PiB ~ αSyn + *APOEε4* + EYO + αSyn*APOEε4, and results are presented on Table 5. The interaction of αSyn*APOEε4 was significant, indicating that the relationship between PiB-retention and αSyn is different in *APOEε4*-positive than in *APOEε4*-negative ADAD mutation carriers. In *APOEε4*-positive ADAD mutation carriers, there was a strong positive association between αSyn and mean cortical PiB (β = 0.39, *p* = 0.032) (Table 5), while this association was not significant in *APOEε4*-negative mutation carriers (β = − 0.10, *p* = 0.348) (data not shown). Exploratory positive associations between αSyn and regional PiB in *APOEε4*-positive ADAD mutation carriers in the 42 regions of interest are illustrated on the 3D brain display, showing strong associations in regions including the superior temporal, anterior and posterior cingulate, parietal and frontal regions (Fig. 9b). The CSF αSyn-*APOEε4*-PiB relationship was specific and could not be replicated by replacing CSF αSyn with CSF t-tau levels (data not shown).

**Fig. 10:**
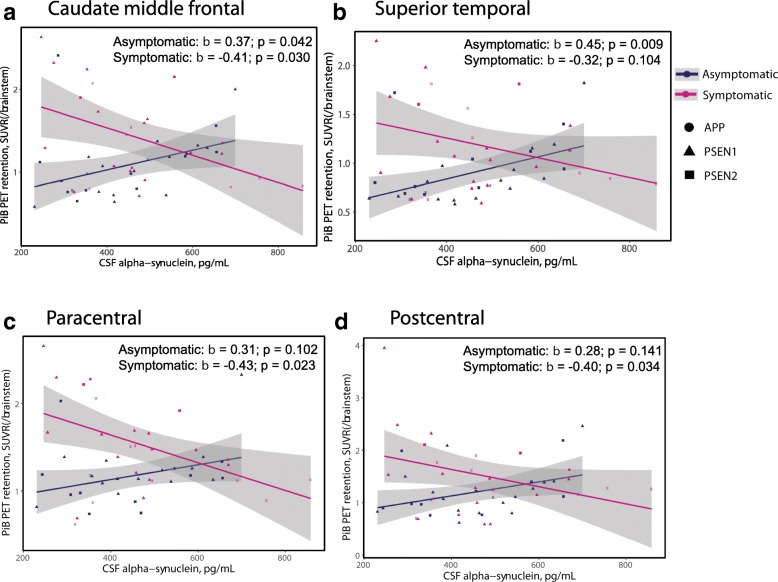
Significant associations between CSF αSyn and regional PiB-retention in PiB-positive asymptomatic (CDR < 0.5, *n* = 28) ADAD mutation carriers (blue) and in symptomatic (CDR ≥0.5, *n* = 25) ADAD mutation carriers (purple) . The significant associations between CSF αSyn and PiB are represented by the standardized β coefficient corresponding to independent predictor αSyn in the linear regression model for PiB ~ αSyn + EYO, where EYO is estimated years from symptom onset, in four brain regions (**a-d**)

The positive significant associations between CSF αSyn and regional amyloid plaque load in PiB-positive asymptomatic ADAD mutation carriers are illustrated in Fig. 10 in regions of early amyloid plaque deposition within the frontal and temporal lobes (Fig. 10a-b), contrasting with respective negative associations observed in few regions in the symptomatic mutation carriers, mostly restricted to areas of late amyloid plaque deposition including paracentral and postcentral regions (Fig. 10c-d).

## Discussion

The role of αSyn in the context of AD pathophysiology is unknown. In this study we set out to investigate the relevance of αSyn to both sporadic and familial AD by examining CSF levels of αSyn and potential associations to various AD disease parameters including cognition, CSF AD biomarkers, and in familial AD patients we also assessed potential relationships between CSF αSyn and brain amyloid plaque burden. Although the significance of CSF αSyn levels to brain parenchymal αSyn levels are still unknown, mainly due to the lack of ante-mortem αSyn brain imaging tools, we anticipate that CSF αSyn fluctuations mirror αSyn alterations in the brain. We studied subjects with MCI, sporadic AD and ADAD in order to pinpoint any potential pre-clinical, prodromal, or mid- to late stage disease pathways linking αSyn to AD pathogenesis. We further explored the impact of the AD risk allele *APOEε*4 on the identified associations.

Numerous studies have reported αSyn pathology in approximately 50–60% of autopsied AD patients [1, 16, 31]. Patients with mixed pathologies as well as animal models expressing combined AD and αSyn pathologies tend to exhibit amplified deterioration, typically enduring more severe symptoms and shorter survival rates [9, 41]. Interestingly, Wirths and colleagues described co-accumulation of αSyn in Aβ plaques and dystrophic neurites only in patients with the Lewy body variant of AD rather than in ‘typical AD’ cases [65]. Furthermore, results from another study of 147 neuropathologically confirmed AD cases suggested that the majority of AD patients who exhibited very few or no neocortical neurofibrillary tangles, termed ‘plaque-only AD’, exhibited Lewy body αSyn pathology, while patients with the Lewy body variant of AD were found to be ‘plaque-only AD’ cases, hence, ‘plaque-only AD’ and Lewy body variant AD were diagnostically indistingushable from one another [17]. Currently, the mechanisms driving comorbid αSyn and AD pathologies have yet to be elucidated making it impossible to predict comorbidity or to diagnose patients ante-mortem, however, we speculate that altered CSF αSyn may be a predictive feature of AD patients that already are harboring or that will develop αSyn co-pathology.

The identification of a molecular interaction between presenilin 1 (PS1), encoded by the *PSEN1* gene, and αSyn was suggested to in part explain the clinical and pathophysiological overlaps between AD and synucleinopathies like DLB [64]. The relevance of this molecular interaction was illustrated by two previous reports demonstrating a strong association between ADAD-causing *PSEN1* mutations and Lewy body pathology specifically in the amygdala [29, 31]. With a direct interaction between αSyn and PS1, the latter of which is a key player in Aβ peptide production [8], it is tempting to speculate that αSyn may have a modulatory role in Aβ production and/or deposition. In support, our current study revealed an association between higher CSF αSyn levels and amyloid plaque burden in several brain areas of PiB-positive asymptomatic ADAD mutation carriers. The brain regions exhibiting the highest rates of annual Aβ accumulation in PiB-negative (< 0.87 SUVR) individuals with pathological CSF Aβ_1–42_ levels (< 192 ng/L), termed as ‘early Aβ accumulators’ by Palmqvist and colleagues in a recent publication [44], exhibited a striking overlap with the brain regions for which we found significant positive correlations between CSF αSyn and PiB-retention. Similarly, a recent study of cognitively normal individuals with subjective memory complaints documented a positive association between CSF αSyn concentrations and brain Aβ deposition [59]. Rather surprisingly, our identified relationship between CSF αSyn levels and Aβ deposition in presymptomatic ADAD patients was not paralleled by any significant correlations between CSF αSyn and Aβ_1–42_ levels. Instead, CSF αSyn levels were consistently positively associated with CSF Aβ_1–40_ levels levels across all studied groups. The relevance of CSF Aβ_1–40_ in the presymptomatic stage of AD, during which Aβ deposition occurs, remains to be determined.

In support of our own results [61, 62] and those reported by other groups [60] we further identified strong positive correlations between CSF αSyn and CSF t-tau and p-tau in both studied cohorts. By stratifying the studied sporadic MCI and AD patients based on pathological CSF AD biomarker levels, we found elevated CSF αSyn levels in patients that exhibited pathological CSF tau but not Aβ levels. Similar to CSF levels of tau, CSF αSyn levels were previously proposed to potentially function as a marker of synapse loss and neurodegeneration [43], however the lack of significantly altered levels of CSF αSyn in AD does not support this notion. A direct link between αSyn and tau pathology was instead suggested by findings showing that αSyn promotes GSK-3β-mediated tau phosphorylation [25]. The authors of the same study further demonstrated that αSyn directly interacted with both tau and GSK-3β. Another in vitro study employing rat PC12 cells demonstrated that extracellular αSyn in fact was involved in GSK-3β-mediated hyperphosphorylation of tau [15]. In cognitively intact control subjects we found strong positive correlations between levels of CSF αSyn and both t-tau and p-tau, which in our view may challenge the notion that the relationship between αSyn and tau is indeed pathological. Due to our findings of a consistent correlation between CSF αSyn and tau levels across diagnostic groups including healthy controls, we speculate that the this association might be due to non-conventional exosome-related release mechanisms [12, 51] for both tau and αSyn, without any clear disease association. The exact relevance of the described seemingly robust relationship between levels of CSF tau and αSyn requires clarification, preferably in future studies assessing potential links between CSF αSyn and ante-mortem tau pathology using novel tau tracers and imaging techniques [50].

With the *APOEε4* allele as a common denominator in terms or risk of disease for both AD and DLB [7] we were interested in assessing potential effects of this gene variant on CSF αSyn levels in the investigated cohorts. In subjects from the MCI-AD diagnostic group who exhibited elevated CSF αSyn levels compared to controls at baseline, homozygous *APOEε4* carriers exhibited the highest CSF αSyn levels. This observation was absent in AD patients and control subjects*.* Hence, we observed an effect of the *APOEε4* variant on CSF αSyn levels in the prodromal phase of sporadic AD, but no effect once patients were clinically diagnosed with AD*.* When considering any effect of the *APOEε4* allele in ADAD mutation carrying DIAN participants*,* we found no differences in CSF αSyn between *APOEε4* positive versus *APOEε4* negative participants, or within the *APP, PSEN1* or *PSEN2* mutation carrying groups. However, presymptomatic Aβ deposition in ADAD mutation carriers was positively associated with CSF αSyn levels only in *APOEε4* positive subjects. We hypothesize that an association between CSF αSyn and Aβ deposition at the presymptomatic stage of AD may be further supported by the *APOEε4* variant which in previous studies has been shown to promote Aβ deposition even in cognitively intact individuals [40].

The regulatory mechanisms governing αSyn levels in brain parenchyma and CSF are unknown. However, there is a clear difference between CSF αSyn levels in AD patients and those with synucleinopathies, where patients afflicted with the latter disorders consistently exhibit reduced levels [18, 24, 38, 39, 56, 57] suggesting a disease-specific process that disrupts the balance between the intracellular and extracellular pools of αSyn. Kallikrein-6, also called neurosin, is one of few reported extracellular proteases shown to cleave αSyn [52, 55]. Increasing the expression of kallikrein-6 in the brains of a mouse model of Lewy body disease promoted αSyn clearance and reduced αSyn pathology [52]. Further, we have shown that patients with synucleinopathies not only exhibited low CSF αSyn levels but also reduced levels of kallikrein-6 [62]. Thus, our previous results combined with those from animal studies suggest that an imbalance between αSyn and kallikrein-6 may promote synucleinopathy. Recently we also reported that the AD and MCI-AD patients examined in the current study did not exhibit altered levels of CSF kallikrein-6 compared to controls, whereas MCI-MCI patients had slightly lower CSF kallikrein-6 levels compared to controls [45]. Hence, the elevated CSF αSyn levels observed in the MCI-AD group were not paralleled by increased kallikrein-6 levels suggesting a potential imbalance between kallikrein-6 and αSyn in this group.

We conclude, CSF αSyn levels alone appear unfit to serve as a diagnostic marker for AD however higher CSF αSyn concentrations were associated with the progression from MCI to sporadic AD, and with the development of symptoms in subjects carrying ADAD mutations. Although not paralleled by significant correlations with CSF Aβ_1–42_, higher levels of CSF αSyn in the presymptomatic stages of ADAD were associated with Aβ plaque burden in several brain regions known to accumulate Aβ pathology during early stages of AD development. The presence of an *APOE*ε4 allele in sporadic AD cases appeared to promote higher CSF αSyn levels which may accelerate the processes linking αSyn to Aβ deposition in AD. The *APOEε4* allele may be involved in molecular processes governing CSF αSyn levels, which in turn appear associated with the presymptomatic build-up of Aβ plaque burden in the brain during AD development. Future studies assessing αSyn in paired CSF and autopsied brain tissues are needed in order to decipher the relevance of altered CSF αSyn levels in the pathophysiology of AD.

## Abbreviations

3T-MRI: 3-Tesla magnetic resonance imaging
AD: Alzheimer’s disease
ADAD: Early-onset autosomal dominant Alzheimer’s disease
ADNI: Alzheimer’s Disease Neuroimaging Initiative
*APOEε4*: Apolipoprotein E epsilon 4
*APP*: Amyloid precursor protein gene
AUC: Area under the curve
Aβ_1–40_: Amyloid-β_1–40_
Aβ_1–42_: Amyloid-β_1–42_;
Aβ_42/40_: Amyloid-β_1–42/1–40_ ratio
CSF: Cerebrospinal fluid
DIAN: Dominantly Inherited Alzheimer’s Network
ELISA: Enzyme-linked immunosorbent assay
EYO: Estimated years from symptom onset
FDG: Fluorodeoxyglucose
MCI: Mild cognitive impairment
MCI-AD: MCI patients who converted to Alzheimer’s disease at the 24-month follow up
MCI-MCI: MCI patients who remained MCI at the 24-month follow up
MMSE: Mini Mental State Examination score
MPRAGE: Magnetization-prepared 180 degrees radio-frequency pulses and rapid gradient-echo sampling
PET: Positron emission tomography
PiB: ^11^C-Pittsburgh Compound-B
PS1: Presenilin 1 protein
*PSEN1*: Presenilin 1 gene
*PSEN2*: Presenilin 2 gene
p-tau: Phosphorylated tau
ROC: Receiver operating characteristic
SUVR: Standardized Uptake Value Ratio
t-tau: Total tau
